# Fair trade in building digital knowledge repositories: the knowledge economy as if researchers mattered

**DOI:** 10.1007/s11019-020-09966-z

**Published:** 2020-07-18

**Authors:** Giovanni De Grandis

**Affiliations:** Department of Philosophy and Religious Studies, Norwegian University of Science and Technology (NTNU), NTNU Dragvoll, 7491 Trondheim, Norway

**Keywords:** Knowledge repositories, Data sharing, Incentives and rewards, Competition in research, Expediential trust

## Abstract

Both a significant body of literature and the case study presented here show that digital knowledge repositories struggle to attract the needed level of data and knowledge contribution that they need to be successful. This happens also to high profile and prestigious initiatives. The paper argues that the reluctance of researchers to contribute can only be understood in light of the highly competitive context in which research careers need to be built nowadays and how this affects researchers’ quality of life. Competition and managerialism limit the discretion of researchers in sharing their results and in donating their working time. A growing corpus of research shows that academic researchers are increasingly overworked and highly stressed. This corroborates the point that the room for undertaking additional tasks with future and uncertain benefits is very limited. The paper thus recommends that promoters of digital knowledge repositories focus on the needs of the researchers who are expected to contribute their knowledge. In order to treat them fairly and to ensure the success of the repositories, knowledge sharing needs to be rewarded so as to improve the working conditions of contributors. In order to help implementing this researcher-centred approach, the paper proposes the idea of expediential trust: rewards for contributing should be such that rational, self-interested researchers would freely decide to contribute their knowledge and effort trusting that this would make them better off.

## Introduction

This Special Issue addresses the question of whether we can trust Digital Knowledge Repositories (henceforth DKRs) in the field of biomedical sciences. An interesting fact about these repositories is that many are envisaged, and many projects launched, but few manage to achieve the volume and uptake needed for taking off as useful research and translational tools or to keep up with the new knowledge constantly published (Baumgartner et al. 2007; Tripathi et al. 2016). So, other questions arise: can we trust that promoters of DKRs will manage to realise their good ideas? Can we trust that a community of experts has the means and motivations for sustaining the growth and continuing update of a new DKR? The risk of failure is high. A lot of attention has been devoted to the risk that *users* may or will not trust the DKRs, and this epistemic trust is surely an important issue. But there is another risk that has been much more neglected: that the knowledge *producers* may not trust that the effort to contribute their knowledge to the DKR is worth their while. I propose the concept of *expediential trust* to identify and draw attention to this phenomenon.

My argument is fairly simple. It starts with the observation that many DKR initiatives are launched relying on unrealistic expectations, because of a wrong assumption about researchers’ motivations and freedom in managing their time. The result is that many attractive projects struggle to be realised and have modest success or fail.

Both existing literature and the case study presented suggest that even very valuable and well-thought projects struggle to win the support of the providers of the knowledge to be collected in the repositories. Indeed, the case study shows that even when the project has the qualities needed to win contributors’ support and shows concern for them, their willingness still cannot be taken for granted.

I argue that the problem of expediential trust is not likely to go away, instead it is actually deeply rooted in the changed circumstances and working environment within which researchers, and especially academic researchers, operate. The entrepreneurial nature of universities and the economic mission of science and research have generated heightened competition, together with a pressing need of a very strategic management of time and objectives by researchers. Endeavours based on altruistic motives or on distant and uncertain rewards can only be supported at a cost and even at a disadvantage. Furthermore, the academic workforce is stressed and strained: growing responsibilities and demands together with increasing job insecurity have skewed the work-life balance. Incentives for the contributors need to be understood in light of the constraints and counterincentives at work in these circumstances, and with a sympathetic eye for the declining well-being of researchers.

I thus suggest that rather than relying on long-term advantages, the *ethos* and values of science, or reputational rewards with uncertain competitive advantages, promoters of DKRs should switch from a view centred on the final product (i.e. on stressing the value the resulting repository will bring about), to a producer-centred view that puts fairness, rewards and sustainability at its core. Taking care of the needs of the intellectual workers who contribute the knowledge needed for the repositories is both the only way to treat them fairly and to maximise the chances of success for the repositories. So, I conclude by urging DKR promoters to make sure that they have the resources to adequately and fairly reward the workforce that is asked to contribute knowledge to the envisaged repositories.

## Background: the problem of the reluctant contributors

Very often the promoters ofDKRs are driven by a compelling view of the possibilities that would be opened up by collecting, structuring, organising, comparing, computing and making easily accessible large amounts of data. This effort is imagined to be able to empower researchers to do things hitherto not possible or extremely time consuming and labour-intensive. New possibilities would open up, more efficiency and speed will be within reach. So, the DKR looks to them as an attractive ideal for a community of users, and since usually among the users there will be those very researchers that are now expected to share their knowledge or data through the repository, the assumption that they will have an effective motivation to do so may seem almost obvious.^1^ After all, bringing about the DKR is in the contributors’ own long-term interest, and as scientists they are believed to be rational people. Furthermore, their contributing is consistent with the practice of sharing that is part of the scientific *ethos*. Logical as it may seem, the reasoning behind this motivational assumption has often failed to stand up to the test of experience.

Bruno Strasser (2017) has studied some of the earlier and higher profile repositories developed in the domain of the biological and biomedical sciences, like the Protein Data Bank and the GenBank, and has found that the attractiveness of the ideal is not sufficient to motivate researchers to contribute their data. He explains the reluctance invoking the *ethos* of experimental scientists, who “valued individual achievement over collective participation”, so that it is particularly challenging to persuade these researchers to share “data difficult to produce and potentially rich in the epistemic rewards of new publications” (Strasser 2017, p. 190). As a result, in the cases he studied, the rate of contributions from scientists was much lower and slower than the promoter had expected and hoped. In order to boost participation and contribution in the endeavour it was necessary to acknowledge the individual need of researchers to achieve recognition for their work through peer-reviewed publication. So, in order to leverage their contributions, the promoters of the repositories had to win the support of the scientific journal editors, who eventually agreed to impose as a condition for accepting submissions that the authors had submitted the data to the relevant repository. Thus, the individual reward had been made conditional to the act of sharing. Therefore, for Strasser, it was not the vision of a collective benefit or an *ethos* of sharing that prompted the contribution of their data, instead “researchers have shared data because it became in their own interest to do so, as defined by the existing reward system in the experimental sciences” (Strasser 2017, p. 198). And that reward system as well as the *ethos* of experimental scientists, Strasser claims, is not communitarian, but individualistic.

What is important for our argument is that Strasser’s case studies show that spontaneous and disinterested contribution was not forthcoming because it was not in line with the existing incentive structure. In order to boost contribution, a targeted alteration of the reward system was needed. Several other studies have confirmed that attempts to promote data sharing continue to meet with limited endorsement from researchers (see Enke et al. 2012; Tenopir et al. 2011 for the results of large surveys). For instance, Hedstrom and Niu (2008), Costello (2009) and Borgman (2012) attempt to understand why in the face of many compelling reasons for data sharing, researchers still resist endorsing sharing practices. All these authors list some of the most important reasons that keep researchers from sharing data, and discuss the problem of lack of incentives and the misalignment between the interests of data users and data producers. However, as it will become clear from our argument, we find more appealing Hedstrom & Niu and Borgman’s approach that stresses the importance of having policies that benefit data producers, rather than Costello’s approach that is oriented to impose data sharing requirements (but he also emphasises the importance of providing resources) and questioning the *ethos* of non-sharing researchers—it is worth noticing that Hedstrom & Niu show that compulsive measures have limited effectiveness and are poorly enforced. Another striking example of the resistance to data sharing is offered by Savage and Vickers (2009) who performed a little experiment and tried to get the data from the authors of papers published in journals that requires data sharing (PLoS Medicine or PLoS Clinical Trials). They tried to contact 10 authors and from the 8 that they could reach, they manage to get the data only from 1.

The importance of the regime of incentives and rewards within which expected contributors work emerged also in the case study that I present in the next two sections below. The significance of this case study is that the project behind the DKR looked like the kind of initiative especially likely to—and quite considerate in the attempt to—win contributors’ support. Yet, in spite of all its qualities, it still run into the challenge of eliciting sufficient contributions and I conclude that uncertainties around researchers’ willingness to contribute is the major challenge to its success.

## The adverse outcome pathway knowledge base as a case study

### The adverse outcome pathway knowledge base

The Adverse Outcome Pathways Knowledge Base is a project originating in the field of toxicology, sponsored by a consortium of organisations including regulatory agencies (like the US Environmental Protection Agency—EPA), scientific societies (like the international Society for Environmental Toxicology And Chemistry—SETAC), intergovernmental agencies (like the EU Joint Research Centre—JRC—and the Organisation for Economic Cooperation and Development—OECD). Adverse Outcome Pathways (AOPs) are descriptions of the chain of biological events leading to an adverse outcome (toxicity) either at the individual level (when human health is at stake) or at the population level (when environmental safety is the concern). These pathways are meant both to systematically collect and organise existing knowledge dispersed in a multitude of studies—as such they are comparable to systematic reviews and other forms of knowledge curation—and to achieve a mechanistic understanding of the biological interactions at various levels of biological organisation (molecular, cellular and all the way up to organisms and populations) and thus they stimulate further experiments and observation in order to fill knowledge gaps. Importantly, the pathway-based outlook dislodges the centrality of animal models, a shift that has profound epistemological and practical implications. In fact, the goal of the project is on the one hand to promote a shift in toxicological knowledge from an observational to a mechanistic mode of knowing (from establishing correlations to understanding causal chains across different levels of biological organisation), and on the other hand to offer regulators and industries an additional tool (not in itself sufficient for risk-assessment) to assess the toxicity of chemicals and reducing animal testing (which is ethically problematic, economically costly and epistemologically not always robust).^2^ The potential usefulness of AOPs would increase as their number grows and an increasing number of biological interactions and relations are mapped and mechanistically understood: this would open the possibility of building predictive models that can reduce or even eliminate the need for animal testing.

It is worth noticing that toxicology and the assessment of chemical substances are an area where many (though not all) believe that major changes are needed and that the current testing practices and methods cannot meet the needs of society. The standard methods of toxicology, according to their critics, are inadequate in many serious respects: too slow to carry out the growing number of needed chemical testing, too expensive, epistemically dubious, ethically under fire because of the heavy use of animals. Many stakeholders agree on the need to develop new and better methods. This widely felt need was addressed by a landmark publication of a large team of researchers under the initiative of the US National Academy of Sciences. The result was a book entitled *Toxicity Testing in the 21st Century: A Vision and a Strategy* (National Research Council 2007).

It is against the background of that vision and of the serious problems that it was trying to address that the AOP-KB has to be seen. The knowledge repository, i.e. the wiki (https://aopwiki.org/), is not the only and final product, but one of the means (together with, for instance, high throughput in vitro system) to achieve a more ambitious goal: the development of computational tools based on a mechanistic understanding of biological processes at different levels of biological complexity. The envisaged computational and predictive tools are not simply an anticipation of future opportunities, but, according to many, the means to overcome a perceived inadequacy of toxicology’s methods. So, contrary to many other cases, the new technology is not coming to disrupt an environment that is functional and stable, but is proposed to a community within which many are dissatisfied with its current tools and wish for a profound renewal. So, the context is such that the promises of the new technology are addressing serious and felt problems. In such circumstances, an opening of trust for promising new technological tools is more likely.

Another important and valued feature of the AOP-KB project is that it has the potential for attracting and integrating multidisciplinary knowledge and to foster collaboration and better understanding across disciplines. This variety of perspectives and skills could also enable a system thinking to emerge from the platform, a possibility that is considered exciting by some researchers. However, making the resource valuable and usable for a broad variety of users is also one of the most significant challenges, as it is shown by the fact that it has struggled to attract contributions from some areas that are perceived to be relevant, like epidemiology and clinical sciences.

### Our study

Between July 2016 and June 2017, we carried out a qualitative study of the AOP Knowledge Base (KB). We conducted 13 semi-structured interviews with members of the community of researchers that have been involved with the AOP KB, both in Europe and the USA. The interviews were recorded, transcribed and analysed through thematic analysis. We also familiarised ourselves with key documents of the community, attended training workshops and participated in workshops aiming to promote the approach, including the 2017 SETAC (Society for Environmental Toxicology and Chemistry) Pellston Workshop dedicated to the AOP approach, which resulted in the publication of a paper (Carusi et al. 2018).

Our main research subject was the AOP wiki, which was at the time the most well-known and utilised tool of the AOP KB project, and that was the instrument for collecting, sharing and updating the various AOPs. The wiki is therefore an example of a DKR. In the course of our research, we were led to pay increasing attention to the factors that may prevent researchers from actually supporting the project to the extent needed for its success. In fact, the desire to better understand the needs of stakeholders and especially of researchers and of accommodating their needs so as to incentivise collaboration was one of the motivations behind the AOP community’s willingness to collaborate with us and support our work. Our case was thus a very interesting one because: (a) is backed by large and reputable organisations, (b) is addressing a need to overcome a crisis in its field, and (c) is promoted by organisations and people who understand the importance of providing incentives to contributors. If the problem of insufficient contribution is affecting even a project with these features, then we take this as evidence that it is indeed a major hurdle for all DKRs and that new ideas, tools and concepts (like expediential trust) to address it are clearly needed.

## The AOP-KB as an example of the challenges faced by promising digital knowledge repositories

### Towards achieving epistemic trust

As a transdisciplinary project that aims to be useful for a broad range of stakeholders—ranging from academic researchers to industry, regulators and decision makers—the AOP-KB needs to meet the epistemic needs of different users. This is clearly a serious challenge and the promoters of the project are making a considerable effort to build a resource that meets the expectations and needs of a broad range of users. However, since my current focus is on the challenge of attracting enough researchers to make DKRs workable, I limit the focus to the demands needed to satisfy the epistemic expectations of the researchers expected to share their data and knowledge. Scientists and researchers expect the epistemic standards that normally obtain in science. Many such criteria have been mentioned in our interviews. For example, AOPs need to be based on experimental evidence, experiments need to be well described and replicable, data sets should be accessible, there needs to be transparency; experimental results are more credible if they are published in peer reviewed journals, if they come from a variety of studies carried out by different research groups; knowledge synthesis should rely on reputable published work, by reputable authors and validated through peer review (Respondents 1, 4, 6, 9, 10). As one of our interviewees explained:

Let’s say someone put something new on the wiki, now this is key, it should be based on scientific work done using models, either in vivo or in vitro, that is well acknowledged, then I would trust it. Now if you can link that, even if it is only in the wiki, eventually people could say, well, this protein has to be activated, if you click on that statement then it would show the experiment or the link to a publication describing those experiments, then I would, you know, tend to believe it, especially if it is done, again, you know, if it is replicable, if other people have done it, it is not just a … strange experiment somewhere (Respondent 4).

A feature that has received special attention by developers and whose importance has been confirmed by many of our responders (Respondents 3, 5, 8, 9, 11) is the rigorous process of peer review to which AOPs deposited in the wiki are subjected. Another important element in building epistemic trust is the fact that the framework and standards of the AOP-KB were produced under the supervision of the OECD, by a reputable team of experts, in a transparent way (Respondents 1 and 3). Overall, the scientific credentials of the projects are generally acknowledged, even though, as it is predictable in such transdisciplinary projects, not everybody is fully convinced by every feature and improvements are suggested. For instance, issues about the external validity of AOPs have been raised (Responders 6 and 7) and it has been pointed out that the computational models are hard to understand for bioscientists (Responder 4). The diversity of users and of their needs has also led to the suggestion (taken up by the developers) of offering different levels of depth and granularity in accessing and visualising the information available through the wiki.

In sum, while there are still some discussions about specific features, disagreements lies at the margins; on the other hand the concept and quality controls at the core of the AOP-KB are generally perceived to meet sound scientific standards and hence the scientific credentials of the project are seen as robust. While this does not solve every issue around the fitness of the AOP-KB for all the practical purposes that it may be expected to serve, I came to believe that the good scientific foundations of the project and the need for developing new testing tools are likely to achieve the needed epistemic trust. Yet, in spite of the urgency of the needs that the new technology is addressing, and notwithstanding the prospect of epistemic trust being within reach, the success of the project is not yet assured. Another challenge repeatedly emerged from our interviews suggesting that epistemic trust is not the only kind of trust crucial to getting the project to work.

### Incentives and sustainability

As part of the vision for a new toxicology, several valuable features of the AOP-KB depend on its ability to contribute to this ambitious vision and of its promise to provide faster, cheaper, more robust and more ethically acceptable toxicity testing methods. This ambition though is conditional on the ability to put together a large and ever-growing pool of AOPs. This is needed:

- in order to work as a one-stop shop for people who need state of the art knowledge,
- in order to enable AOP networks,
- in order to avoid unnecessary duplication of results,
- in order to become a system thinking tool,
- and, most fundamentally, in order to develop predictive computational models—the vision for a new toxicology.

This leads to a crucial practical question, i.e. whether researchers and organisations have enough motivation and incentives for contributing their knowledge to building new AOPs according to the set protocols and uploading them in the wiki. This is a necessary condition for the success of the project.

Researchers have mentioned a number of interesting reasons for supporting the AOP-KB and contributing to it: contributing to the reduction of animal testing (Respondents 1, 2, 12, 13), hoping to expand professional networks (Respondents 2, 9), hoping to give more visibility for one’s research (Respondent 4), getting validation or recognition of one’s research (for those working outside academic science) (Respondents 2, 5, 8), supporting a tool and a project in which they believe and have invested energy (Respondent 9), pursuing the ethos of scientific sharing and altruism (Respondents 4, 9, 13).

But in spite of all the reasons above, winning people’s active involvement and commitment appears to be the most serious challenge. As two respondents (7 and 4) put it:

we have noticed that it's difficult to engage research organisations to bring their knowledge to our KB because, what's in it for them?

It’s very difficult to get people motivated to work on the AOPs.

The analysis of our interviews helped to articulate the challenge and identified 3 key facts around which there was broad consensus.

1. Most scientists (especially those in academia) need to give priority to journal publications over depositing an AOP in the AOP-KB (Respondents 1, 4, 6, 7, 8, 10, 11, 12, and 13) “Because”—Respondent 4 explained—“we are evaluated on publication, not on what we put on a website” [see also R10].
2. Depositing an AOP on the wiki is considerably time consuming and does not bring with it enough immediate rewards to motivate many and thus more or new incentives are needed (Respondents 1, 4, 5, 7, 8, 9, 11, 12, 13); and it is sometimes seen as being in tension with the traditional incentives of publication and citation (Respondent 11).
3. Many responders suggested that the actual success and uptake of the AOP-KB by its intended users, especially regulators and policy makers, would be an important motivator. Examples of use and success stories could be a powerful incentive (Respondents 2, 4, 8, 9, 10, 13) both because they would show that the effort has a point and because it could substantiate the claim that contributing to the AOP-KB is a significant way of having societal impact.

People need to see that important decisions are being made on it, or it helps them to do what they couldn't do before, that will really help. Because it's a fair amount of work to put a full adverse outcome in there (Respondent 8).

Our findings show that providing incentives to researchers who can contribute to the building, reviewing and updating of AOPs in the KB is a problem that has to be tackled and solved.

### The way forward

The results of our analysis and further engagement with the promoters of the AOPs-KB project led us to believe that securing an adequate and sustainable stream of AOPs and of updates to the KB cannot be achieved with one simple solution. It requires an articulated strategy that operates at different levels including improving the usability and usefulness of the resource (both for contributors and other stakeholders), a much more formalised system of recognition for contributors at various levels (and better coordination and synergies with scientific journals), the introduction of financial compensation for the time spent feeding the AOP-KB, and finally some cultural-organisational transformations so that curation work is supported by the relevant research organisations and at the level of science policy.

These challenges can be described in terms of trust. Epistemic trust needs to spread wider, trust needs to grow around fittingness for practical purposes and most of all trust needs to be built on the fact that it pays off to contribute to it. Even though the promoters of the AOP-KB have attempted to make contributions acknowledged and the standards consistent with those needed to build scientific reputation, the incentives are not yet so compelling as to ensure the required volume of growth and the long-term sustainability and further initiatives and resources are needed, as representatives of the AOP community acknowledge (see for instance Carusi et al. 2018). Clearly, the responsibility cannot rest on the promoters of DKRs alone: funders need to make more resources available and stakeholders need to act in concert in order to make the institutional changes needed to support projects that take seriously their own sustainability and the needs of knowledge contributors.

## My proposal: focusing on knowledge producers through the idea of expediential trust

The persistent difficulty that DKR promoters encounter in recruiting contributors persuaded me that new perspectives and ideas are needed. At the same time my experience in academia—where I have witnessed how heavy workloads and career demands can be—suggested to me that responsiveness to contributors needs and well-being is both ethically required and instrumental for DKRs success. So, I propose a knowledge producer centred approach.

Rather than sticking to Strasser’s hypothesis about the individualistic ethos of the experimental scientist, I focus on the structural changes in the institutions, drivers and political economy of knowledge production, and on the impact that they have had on the life and wellbeing of researchers. Building on an extensive corpus of research on the changing nature of scientific research and institutions, I formulate the hypothesis that such profound changes have introduced tight constraints to what researchers can do without suffering a competitive disadvantage or taking up burdensome and hardly sustainable additional responsibilities. I do not take any moralistic attitude towards researchers and their motivation, on the contrary, building on a growing body of evidence of the strains and stress suffered by academics, I take a sympathetic look at the pressures and demands under which they currently work. Taking their struggles and needs seriously, I advocate the importance of fair working practices (or production processes) around knowledge sharing—a kind of fair trade approach for workers in the knowledge economy.

At the level of individual motivation, it is a well-established principle of many coaching and counselling techniques to recommend to people to get a taste for the process that leads to a result, rather than trying to be motivated by the lure of the distant goal in order to endure current and forthcoming sacrifices. The distant goal is too remote a reward, hence, to sustain an effort, some current benefit is needed. If we adapt this model to data sharing, we need to make some adjustment and not simply expect that researchers will come to like the work required by data sharing—as Hedstrom and Niu (2008) observe, “it is hard to imagine how to make it fun to create ‘archive ready’ data”—but rather take steps to make sure that they will receive some external benefit (extrinsic rewards) from incorporating that work in their practices. With this kind of adjustment, I believe that this approach should be applied to the production of DKRs. Namely, promoters of the idea should abandon the final endpoint attraction strategy (the gravitational pull model, so to speak), and instead adopt a producer-centred perspective, and a generous one, inspired by the ideals of fair trade: data are a product that should be valued and rewarded adequately and some provision to improve the working life of the producers should be put in place. The knowledge economy has been touted as something good because it would generate highly qualified and attractive jobs, but, as I show in Sect. 7 below, the reality is that the quality of life and even the mental health of knowledge workers is steadily deteriorating. Increased demand for knowledge has not brought benefits to the workers supplying that knowledge, but only added weight to their workload. With this concern in mind, I propose the concept of *Expediential Trust* as both a useful analytical and practical tool. Expediential trust helps in understanding not how to compel people to contribute, but how to make it safe and rewarding for researchers to contribute. So, the concept of *expediential trust* is proposed as a tool, a fictional device to help in setting up a *fair and sustainable process of knowledge sharing*.

The concept of expediential trust is meant to describe the kind of trust that is needed to sustain a commitment and effort from people who have to manage their time and workload in light of strict targets, accountability to their employers and tight career management demands. It is proposed as complementary to the concept of epistemic trust. While this latter focuses on the epistemic requirements that are needed to win the approval and trust of scientists and other professional experts, the idea of expediential trust points to the incentives and bonuses that experts and scientists need to have in order to endorse a project that makes new demands on their time and efforts. Let me try to make this distinction as clear as possible. Kankanhalli and colleagues have noted that.

Success of EKRs [Electronic Knowledge Repositories] requires that knowledge contributors be willing to part from their knowledge and knowledge seekers be willing to reuse the codified knowledge (Kankanhalli et al. 2005, p. 115).

Epistemic trust looks at the conditions needed for knowledge seekers to be willing to use the knowledge offered, while expediential trust looks at the conditions needed for knowledge contributors to be willing to make their knowledge digitally shareable.

But what kind of trust is expediential trust? It is clearly not a default and pre-reflective disposition, as the trust described in many accounts of social or interpersonal trust (e.g. Luhmann 2017), but rather an explicit type of trust, emerging from reflective awareness of facing a choice. In this respect, it is closer to the philosophical accounts of trust that ask whether trust is well-founded, but rather than focusing on the cognitive and epistemic element, it revolves around the expected payoffs. So, it is triggered not by an epistemic doubt: “shall I trust this belief or report?”, but by an expediency doubt towards a collective endeavour: “shall I trust that joining a collective effort will pay off?” (an analogy can be provided by the choice of a pension scheme or health insurance, or whether to join a housing cooperative). It is a question that arises when there is a time gap between a demand for a contribution now, and the expectation for a reward later, provided that other people too have contributed to the collaborative scheme and made it viable and sustainable. Contrary to some basic social practices, where the cost of opting out is so great that people trust by default—because it is too costly to distrust—the question of expediential trust emerges when not joining a collective endeavour is clearly a practicable option, hence joining involves a risk and reasons to take it. This kind of trust can be analytically divided into a technical and an ethical component. We may question whether the collaborative scheme is well designed and suitable to deliver the expected result, and we may also question whether the other participants in the collaborative scheme will support it consistently and dependably. Furthermore, there is an additional ethical dimension: is the scheme distributing burdens, risks and rewards fairly? This is the central concern at the basis of the idea of expediential trust. By postulating that people can safely keep out of the scheme, expediential trust invites us to frame the choice so that it has to be attractive and fair for people who join the collaborative effort. People should join because they trust that the collaboration is designed in such a way that it makes their condition better than their current one, instead of joining because they are afraid of being left behind or penalised if they do not join, which is the mechanism used by the compulsory policies.

However, the most important thing is that the concept is *not* proposed as a heuristic tool to be used by the people who are facing the actual choice, namely the researchers. Rather, it is proposed as a kind of *thought experiment*, as an intellectual device to help the imagination of those who are shaping the practice: how do we need to design the practice, its rules, demands and rewards so that people evaluating the practice will be willing to join and sustain it? Given that John Rawls’ fiction of the original position (Rawls 1999) is so well known, it can be used to illustrate, by analogy, the role that I attribute to the concept of expediential trust. Rawls proposes the idea of the original position as a fictional choice, as a device of representation, i.e. as a test for assessing the fairness of principles of justice for the organisation of society. If we want to work out fair principles for society, we need to design them so that they will be chosen by hypothetical citizens who are self-interested but that they do not know their position in society. Rawls has very often been misunderstood as claiming that real people are like people in the original position, but this is a misreading. Expediential trust is proposed to perform an analogous function: it is meant to help to design collaborative schemes for knowledge sharing that hypothetical researchers free to choose whether to join or not would find appealing and rewarding. So, the concept is addressed not to real researchers (and neither assumes that they are self-interested nor recommends that they should be), but to those designing the schemes for knowledge sharing, with their demands and incentives. Real researchers are hopefully motivated by other expectations, for instance the desire to participate in an epistemic community and support it.

The concept of expediential trust is meant to help those shaping science policy and knowledge-sharing projects to design their plans as if they could win expediential trust from researchers. By doing so, we expect them to achieve two results. First, they can better achieve their goal by winning researchers’ compliance. Second, they can achieve their goal not through imposing additional burdens to the producers of knowledge, but by making their working life better. Thinking in terms of expediential trust can make knowledge-sharing policies and projects both more successful and more ethical. To wit and paraphrase the subtitle of Schumacher’s (1973) famous book (*Small is beautiful. Economics as if people mattered*), the motto behind expediential trust and fair knowledge sharing would be “the knowledge economy as if researchers mattered”.

Raising the question of expediential trust does not mean claiming that researchers are only moved by self-interest, that they are textbook examples of *homo economicus*. Nothing could be further from my intentions. Rather, *the question of expediential trust leads us to ask whether researchers can afford to take up new and demanding commitments when their duties are tightly specified and when they are at risk of being out of the research game if they do not manage their priorities and efforts strategically and efficiently.* The successful realisation of DKRs needs to be, and to be trustfully perceived to be, in the interest of those researchers at the coalface, or, if you prefer, at the shopfloor level. Just like administrative reforms will not work if they do not win the support of street level bureaucrats, lofty scientific ideals will not be achieved unless researchers believe that it is wise and sustainable for them to put work and time in their pursuit. The transformation of research management forces DKRs promoters to think in terms of expediential trust, lest they pursue illusory and futile goals. Furthermore, the deterioration of the wellbeing of academic researchers prompts DKRs promoters to think in terms of expediential trust, lest they want to achieve their goal through making more miserable the life of their knowledge providers.

The usefulness of expediential trust emerges from a tension at the intersection between policies that promote knowledge sharing and the current governance and management of research—one expects generosity and disinterestedness, the other promotes single-minded efficiency and productivity. When the ideal of sharing knowledge through digital repositories is pursued relying too much on the traditional understanding of the *ethos* of science (understood as including disinterestedness, commitment to knowledge and to sharing it) the actual constraints under which researchers currently work are overlooked. As a result, the realisation of the ideal is based on an idealisation of real circumstances rather than on a sound appraisal of the resources needed. The concept of expediential trust is proposed as a reminder that the active collaboration of researchers neither depends only on having a goal that satisfies the epistemological standards of scientists (i.e. on earning epistemic trust), nor should be achieved through imposing new obligations on researchers and shaming those who resist the sharing practices.

Can we instead pursue better science and increased societal benefits while making the life of knowledge producers better rather than more difficult? Our point is simply that techno-moral change (Swierstra 2013)—i.e. change in practices and moral judgments that are partly triggered by adopting new technologies—that looks desirable comes with some costs. Ultimately these costs are borne by people, and this raises two important questions: are these costs too high and imposing unjustifiable misery on some people? Are the costs distributed fairly?

## Knowledge sharing and the managerialisation of research and innovation

In this section I argue that the current institutional circumstances within which research is carried out generate a structural misalignment between the expected sharing *ethos* of scientists and the structure of incentives under which they work. A consequence of this misalignment is that knowledge sharing may be promoted counting on an incomplete and partially outdated understanding of the *ethos* of researchers and of their current working circumstances. This could result in policies and strategies that disregard the needs and constraints under which researchers work, impose new burdens on them and even point a moralising finger at them.^3^ To prevent these outcomes, I have proposed the concept of expediential trust as a thinking device for imagining the conditions under which researchers’ collaboration would be voluntary and fair.

The transformation of science and research have been widely studied and discussed and one thing about which there is very substantial consensus is that science and research have been practiced under rather different institutional circumstances in the early decades after the war and in the last decades. Here I focus on universities in particular, although of course not all researchers work in academia. However, academic researchers represent a very substantial group, and some of the transformations described (like increased competition and strict accountability regimes) apply widely across the research institutions. What changed most obviously were the arrangement and management of the research organisations: their mission, funding and accountability; as a result, the practices, the *ethos*, the incentives and career structure profoundly changed as well. John Ziman in his Book *Prometheus Bound: Science in a Dynamic Steady State* (Ziman 1994) tried to convey the extent of these changes through the thought experiment of imagining a researcher coming back to her department after 30 years of space–time travel and notes how she would be exposed to a number of new concerns and worries that are now prominent in the mind and talks of her colleagues: from the attempt to align with R&D policies and priorities to how to compete for scarce resources, from concerns about performance indicators to the need of managing a portfolio of skills (teaching, research, outreach, administrative, fundraising, collaborative, leadership are commonly expected skills from applicants for academic jobs or research funds) in order to secure the next, non-permanent job and so on and so forth. Ziman concludes that.

In less than a generation we have witnessed a *radical*, *irreversible*, *world-wide transformation* in the way that science is organized and performed (Ziman 1994, p. 7).

Furthermore, he stresses how these changes have penetrated deeply into the daily practices and the culture of research, to the effect that they are now part of “the *climate* in which scientists have to live” (Ziman 1994, p. 5). For our current purposes we do not need to be very precise about when the transition took place—most likely these changes started in the late Sixties, accelerated in the Seventies and Eighties and were globally well established by the mid 1990s.^4^

What is important for my argument is that we now live at a time when the values and culture of research suffer from a strange form of dissociation between the lived experience of researchers caught between the pressure (and necessity) to achieve their contractual goals (deliverables), boost their scientific production, expand their network, carefully plan and manage their career and daily schedule on one hand, and on the other hand a dominant rhetoric still powerfully influenced by the scientific culture and values that prevailed before the recent institutional transformation. This is not only because often cultural change is slow and there are survivals, but also because in many ways those values are still compelling and attractive. I see colleagues (and myself) motivated by and committed to these values—although these motivations and commitments are bounded by a grid of external constraints and new interiorised goals and values. But these values and ideals are no longer the only and dominant defining values of researchers. Researchers are often experiencing a painful value schizophrenia: to put it bluntly they have both a scholarly, idealistic-perfectionist ideal and an entrepreneurial, efficiency-productivity ideal. They are moved by curiosity and by the desire of deploying all their skill and ingenuity in their scientific activity and production, by the desire to do it to perfection; but, at the same time, they want to incarnate and express those broader skills that make for a successful researcher. They want this not only because of ambition, but because the profile of a successful researcher under current circumstances is a fascinating ideal too. It requires a very complex set of skills and it can appear more complex, balanced and less obsessive than the purely scholarly ideal.

So, here we are seeing neither a conflict between “pure” scholarly values and “corrupted” scholarly values, nor a conflict between the smugness of ivory tower scholars and the pragmatic scientist engaged in the real world. Both ideals are respectable at their best and subject to excess or degeneration. What is important is that their coexistence creates tension and sometimes irreconcilable demands. Sometimes a satisfying and sustainable compromise can be found, sometimes one ideal needs to be—temporarily, it is hoped—forfeited.

However, it has to be acknowledged that the older set of scholarly values are often perceived as loftier, nobler and purer. Furthermore, they are associated with the dominant scientific *ethos* of the times when science established itself as a driving force of modern society and as the most respected source of authoritative knowledge. Arguably, they are also needed to support the prestige and credibility of science. The noble ideals and the virtues needed to realise them create an ethical image of science practitioners and hence of the practice of science itself. Conversely, the new circumstances of research do not seem to have been accompanied by an ability to articulate a new scientific *ethos* that can be seen as equally noble, compelling and distinctive. Moreover, the perception that traditional values are still obtaining, represents an asset also for achieving the new mission of research.

However, while in practice researchers have to find an integration, or at least an accommodation, between the old aspiration and the new demands for efficiency and usefulness, at a theoretical level we still have to see a new formulation of the new key values and norms of science that could match the attractiveness and respectability of, say, the Mertonian account.

But what are these institutional transformations we are talking about? The transformations most relevant to our argument are the following: the belief that research plays a vital role in sustaining the success and competitive edge of a country and of its industrial and economic productivity (Etzkowitz et al. 1998; Gibbons et al. 1994; Rossi 2009; Zawdie 2010)—this is often described as the “third mission” (after teaching and research) of universities; from this follows the belief that policy should support research to the extent that research reorients its mission towards economic development and social impact (Guston 2000; Guston and Keniston 1994; Marburger 2015; Ziman 1994)^5^; another important point is that resources allocated to science and research cannot be assumed to be growing and during hard times are subject to cuts, so research is now operating under circumstances of resource scarcity and competition (Sarewitz 1996; Ziman 1994); taken together the previous points lead to the further corollary that the allocation of resources for research will be largely based on research projects’ or research groups’ ability to provide evidence of their impact on economic performance or their contribution to social goals—like security, public health, natural resource management—and to enhancing citizens skills and lifelong learning, cultural development and knowledge-based development (OECD 2007). Finally, these changes have forced universities and other research institutions to improve their efficiency and competitiveness through adopting managerial techniques coming from business, so much so that the concept of “entrepreneurial university” has become quite popular (Etzkowitz et al. 2000; Foss and Gibson 2015; Gibb and Hannon 2006; Guerrero and Urbano 2012; Meissner et al. 2018). University are, as an OECD report puts it, “in the knowledge business, which is a competitive and fast-moving sector” (OECD 2007, p. 13). An implication of this new mission and organisational model is that they need to be “entrepreneurial at all university levels” (Guerrero and Urbano 2012, p. 54), and this of course effects a transformation of the organisation’s culture, of its *ethos*. It also implies that efficiency becomes a key organisational criterion: to be drivers of innovation and growth universities need to be on top of the game and therefore they need to use their resources, including their human resources as efficiently as possible. A further corollary is a culture of performance indicators, which is of course applied to the performance of teachers and researchers. Needless to say, not all researchers operate in universities, but these entrepreneurial and efficiency demands apply as well in industry and in many—although not in all—other research institutions.

This description should provide a context that helps in explaining why researchers may find it hard to contribute to knowledge repositories if their working contribution is expected only on the basis of the traditional scientific value of knowledge sharing (what Merton (1973) called “communism” and other authors following him renamed “communalism”). Many researchers do see the appeal of that value and are inclined to respond to it, but they may not do so because they are constrained by tight demands and pressures stemming from the insecurity of their position or funding. When their time is carefully managed in order to perform their obligations, adding another time-consuming service may simply be too much: something that they cannot sustainably squeeze in. Similarly, when competition for jobs and funding is very intense and both resources are scarce (as Nikunen (2012) effectively puts it: “The number and supply of researchers is high, while secure positions are scarce”), to devote time and energy to something that your colleagues/competitors are not obligated to undertake may put you at risk of suffering a competitive disadvantage that can drive you out of the game. I thus suggest that both the success of knowledge sharing initiatives and concerns about the fairness of the distribution of the burdens necessary to sustain it demand that the question of expediential trust is heeded and that strategies for generating it are developed.

## The deteriorating working conditions in academia

An academic career was once viewed as offering low stress, secure, safe employment, and high social standing with opportunities to do satisfying, autonomous work (…). Over the past 20 years, the academic environment and perceptions about academic careers, have changed drastically (Catano et al. 2010).

The transformation described in the previous section has not only limited researchers’ discretion in using their time and their liberty to devote themselves to disinterested and altruistic pursuits, it has also profoundly affected their working conditions, well-being and mental health. A growing body of research and of public personal testimonies (Dunn 2013; “Quit Lit,” 2019) show very clearly how serious these trends are. The managerialisation described above—with its increased competition and drive towards efficiency and performance assessment—has contributed to well-documented rising levels of stress and anxiety among academics (Abouserie 1996; Berg, Huijbens, and Larsen 2016; Biron, Brun, and Ivers 2008; Gill 2010; Gillespie et al. 2001; Kinman 2001; Tytherleigh et al. 2005; Winefield et al. 2003, 2008), to declining levels of job satisfaction and inability to achieve a satisfying work-life or work-family balance (Bothwell 2018; Cownie 2004; Kinman and Jones 2008; Pillay and Abhayawansa 2014; Slišković and Seršić 2011), to overwork and burnout (Hogan, Hogan, and Hodgins 2016; Watts and Robertson 2011), to widespread feelings of inadequacy and fear about the future (Ball 2015; Gill 2010; Morrish 2019), to rising levels of mental health problems (Gorczynski 2018; Kataoka et al. 2014; Kinman 2001; Mullings, Peake, and Parizeau 2016; Pace et al. 2019). These results consistently emerge from research carried out since the 1990s in the UK and Europe, North America, Australia and New Zealand. The most frequently mentioned causes for these alarming outcomes are work overload, excessive administrative tasks, insufficient resources, job insecurity, intense competition, and insufficient recognition and rewards.

Once this picture is used as a background for understanding the deep causes of the limited endorsement of the sharing practices required by contributing to the DKRs, things begin to make sense. Researchers feel that they have work overload and that they are struggling to cope with it, and the main obstacle that they report to contributing to DKRs is that it is time demanding (Tenopir et al. 2011). This cannot be surprising. Researchers report distress and anxiety related to competition and job insecurity and another main reason that they report for not contributing to DKRs is the perceived necessity to prioritise their publications and making the most of their data (Borgman 2010; Hedstrom and Niu 2008). Again, this seems perfectly logical. Just as these causal links are obvious, I believe that it is evident that any strategy to increase participation in the sharing practices required by DKRs based only on compulsion, shaming or hypothetical future reputational gains are not only inadequate but insensitive, if not callous and exploitative. Instead, I propose to echo the suggestion made by Pace and colleagues to improve the quality of academic work and argue that efforts to improve sharing “that simply ask academics to do more without reference to their primary work motives” and duties or to the reality of their daily practices and struggles are bound to fail. Only a fair allocation of workload will enable researchers to work better and reduce their psychological distress (Pace et al. 2019). Furthermore, I endorse Morrish’s admonition that “the simple, humane values of kindness and care for each other must be recovered if we are to ameliorate the toxic university” (Morrish 2019).

An idea that was suggested by the promoters of the AOP-KB was that of buying-out the researchers’ time needed to share their knowledge and data. I believe that this points in the right direction, because it represents a genuine departure from the strategies leveraging on compulsion and reputation. Buying researchers’ time is a real and immediate reward, that shows an appropriate valuation and appreciation of researchers’ time and effort. This is in stark contrast with the reputational rewards, which, under the current conditions described above, are reinforcing a logic of relentless competition that makes people overworked, stressed and miserable.

If organisations really believe in the value of data and knowledge, then they should commit the resources needed to produce them, without expecting that they are simply brought about through the motivations of an economy of insecurity and hope. What the reputation-centred strategy fails to appreciate is that in a intensively competitive environment introducing a further scientific productivity metric for building a researcher’s reputation is imposing a burden on researchers, while it is not giving them a direct reward, but only a competitive edge that will disappear once the practice has become a baseline professional requirement. At that point there will be no competitive advantage in contributing, but on the other hand the task will have become an additional standard duty, without any certainty that the additional time and effort that it requires will have been recognised as part of the workload of every individual researcher. To be sure, at this point researchers will have access to powerful instruments that will make their work more productive and possibly also more rewarding, but this increase in productivity will benefit many other stakeholders, which should bear some of the cost of achieving this productivity gain.

Suppose that in some manufacturing industry workers were told that they should work five extra hours per week without retribution to contribute to the optimisation of the production line. Once this will be achieved, there will be a substantial gain in productivity—which will benefit the industry and its customers—and the workers will have the satisfaction of seeing that they will achieve more during their working hours (but they will have to keep working additional hours to keep updated the new system). Who would expect the workers and their trade unions to think that this is a good deal and they will benefit from it? We would surely expect that they would accept only under one of the following scenarios: (a) they get paid at a good rate for the extra hours; (b) they do perform the new task during their normal working hours, not as extra hours; (c) they are threatened to lose their job if they do not accept the deal: the company is ready to relocate into a lower income area where workers will accept to work the additional hours for the same salary. Now, unfortunately leveraging only on the reputational incentive bear no resemblance with options a) and b), but only with option c): because if researchers do not accept, they will suffer the competition from researchers who are prepared to do some extra and unrewarded work. Proposing such a deal to the workers surely does not look like kindness and care.

The digital revolution has also shown us the dangers of relying on voluntary contributions and reputational hopes: it is a way of mobilising many people and pushing them to contribute and to share in the expectation that this will earn them a reputation that in the future will pay off. In this way, their free contribution crowds out the work of paid professional who were providing the same services for a present and tangible reward. Actual rewards are replaced by virtual and hypothetical rewards. Professional performances provided for a fee are replaced by tasks carried out in pursuit of possible future advantages. In the case of DKRs, since they are relatively new kinds of goods, they could not rely on an existing dedicated profession devoted to them, so there is not the crowding out effect: we do not have professionals being replaced by amateurs hoping to become free-lance content providers. Yet, we still have unpaid (highly qualified) service provision expected instead of paid-for service provision—and that is why buying researchers’ time for doing that would be a paradigm shift. On the contrary, the reputational reward feeds the toxic trend of expecting academics to continually expand their skill set and their achievement record at an unsustainable pace. As Berg and colleagues note,

academics must now constantly seek ways to increase their future value, through, for example, successful grant applications, peer reviewed publications (in journals with the “right” impact factor), website blog posts, hits on their personalized socio-scholarly media websites (…), paper citations (…), and various other “measures of esteem” (Berg, Huijbens, and Larsen 2016, p. 178).

This is precisely one of the key drivers of researchers’ stress, anxiety and mental health problems. Can we really blame those among us who resist these pressures to do additional work for free, to give our scarce and precious time, to “fabricate ourselves in ever lengthier and more sophisticated CVs” (Ball 2015, p. 259), who answer, like Melville’s Bartleby: “I would prefer not to”?

## Conclusion

There is a puzzle around DKRs: everybody seems to agree that they are very attractive resources and that it would be good to build them, yet a lot of the attempts to realize them lose steam and fail. Like every human and collective endeavour, they can fail for a number of reasons, ranging from lack of resources, poor planning, bad luck or timing. From the perspective of a philosophical analysis of disruptive digital technologies, attention has mainly focused on epistemological issues—and more specifically on epistemic trust and on the transformations brought about by aggregating and standardising knowledge through the support of digital technologies—and the new social network that they create—epistemic communities. Our experience with the case of a quite high-profile digital repository in the field of toxicology, the Adverse Outcome Pathways Knowledge Base, shows that indeed epistemic trust is of crucial importance and a necessary condition for the success of these kinds of projects. However, interviews and engagement with the AOP community revealed that another serious challenge could prevent the success of the initiative: the struggle to attract enough contributors and to keep them committed to supporting the growth of the Knowledge Base. This challenge led me to articulate the notion of expediential trust to help focusing attention on the needs of the researchers who feed knowledge into the repositories. The work needed to support the creation of knowledge repositories is very often voluntary work, done out of an altruistic commitment to create a resource valuable for the research community to which researchers belong or aspire to join. While I do not believe that this motivation has lost its appeal, I think that it may be too remote when challenged by powerful competing demands and counterincentives. Thus, expediential trust attempts to use instrumental rationality to pursue communicative rationality or reasonable fairness—under the guise of conditions of work that would be acceptable for unconstrained rational agents.

From the perspective of the researchers who are supposed to contribute content and curation services to the DKRs, this additional task has to be compatible and sustainable from two different perspectives: (1) from a personal ecology of practices, and from (2) an interpersonal reality of competition for scarce resources. So, before committing to contributing time, work and research findings to a knowledge repository, researchers are bound to ask themselves: is this further commitment to do extra work and share my research results something that is sustainable? Is it contributing to my successfully performing my professional targets and goals? Is it a practice that can be sustainably integrated into my working routine or is it further eroding my already limited workfree time? Is devoting time to the task and sharing my knowledge going to be a competitive disadvantage in my quest for jobs, progression, funding? These, we believe, are questions that is perfectly reasonable for them to ask in the current research and employment conditions. But acknowledging that these are sensible questions for them to ask means that promoters of DKRs need to consider them too, because the support of knowledge producers is necessary for the successful development of DKRs. The promoters are therefore facing an alternative: they can either look only at the result they want to achieve, or they can look also at the impact of their initiative on the people who can make it work. I believe that the latter approach is both instrumentally more effective and the only ethical approach that looks at researchers not merely as means—human resources from which to extract value—but also as ends in themselves, as people whose life can be made better or worse. I therefore urge promoters of DKRs to use the concept of expediential trust in order to adopt the point of view of researchers and take into account their actual working conditions.

If promoters want to raise the chances that scientists share their data, they should better ask themselves what kind of support, compensations and rewards will make it possible for them to share, while keeping their workload manageable, and their life-work balance healthy and sustainable, while achieving their professional goals and not losing competitive advantage. In the past decades we have seen digital technologies bringing many advantages to users while making the conditions for the providers of goods and services worse. Surely, we do not want science to do that to scientists, especially if we believe in the strategic importance of knowledge and that “a motivated academic workforce, satisfied with their reconstructed academic jobs, is most likely to produce the greatest benefit to research, innovation and society” (Bentley et al. 2013, 240). Perhaps it is time to turn on its head the famous exhortation by president Kennedy^6^ and not to ask what scientists can do for science, but what science policy and scientific projects can do for scientists as workers already experiencing heightened demands and declining well-being. If we want to do science as if researchers mattered, then to think in terms of expediential trust when we design digital repositories would help.

## Footnotes

1 In fact, the assumption is simplistic, for researchers may at the same time want to access other researchers’ data and protect their own data. “Scientists would like access to everyone else’s data though they do not necessarily wish to reciprocate” (Walker, quoted in Strasser 2017, p. 194). “Researchers’ incentives to release their own data may or may not align with their motivations to gain access to the data of others” (Borgman 2012, p. 1067).

2 Once a chemical is shown to be capable of causing the molecular event triggering an AOP, knowledge of the pathway enables to anticipate consequences at higher levels of biological organisation. It is important to stress that AOPs are not chemical specific, so that they can be relevant for assessing multiple chemicals: in fact, all those that lead to the Molecular Initiating Event (MIE) which represents the starting point of an AOP. This also helps to understand that while the building of AOPs cannot currently do without animal testing, still it can greatly contribute to reducing the need for them: if a chemical triggers a causal pathway that leads to an adverse outcome, there is no need to demonstrate every time anew that the causal chain will lead to the adverse event. Here is where knowledge of causal mechanisms replaces observation of effects in whole animal organisms.

3 Consider for instance the following statement by Costello (2009, p. 424): “Withholding data after they have been … published with the intention of professionally profiting further, raises ethical concerns about whether the scientist is really motivated to advance science”. I suggest that in our liquid times (Bauman 2007) even scientist may be concerned first of all to stay afloat, and I would be cautious before casting the first stone.

4 “Twenty-five years ago the dominant imagery of the relationship of science to society at the National Science Foundation (NSF) in the United States, for example, was one of protecting basic research; the image was one of being at the frontier. In the 1990s the dominant image in the same funding and opinion-leading agency is what a couple of science studies colleagues call “engineering the cash crops” of marketable results of science. The attempt is to build marketability into the research system through an external ethic, or ethos” (Elzinga 1997, p. 424). “From the early 1980s onward, the policies and priorities of universities have been increasingly influenced both by the quest for nationally relevant university research and by the pressure for accountability and cost reduction” (Geuna 2001, p. 609).

5 Marburger, who was G.W. Bush science advisor, was strongly committed to find better econometric indicators for evaluating investment in basic research and when he tried to get some suggestion from the economist Ben Bernanke (who was later to become chair of the U.S. Federal Reserve) all that he got was the blunt reply that he did not “think that the government should be funding anything that does not have demonstrable economic consequences” (Marburger 2015, p. 209). The imperative for science to show impact can hardly be put more starkly!

6 “Ask not what your country can do for you—ask what you can do for your country”.
